# Quality control of the traditional Chinese medicine Ruyi jinhuang powder based on high-throughput sequencing and real-time PCR

**DOI:** 10.1038/s41598-018-26520-3

**Published:** 2018-05-29

**Authors:** Qiang Li, Ying Sun, Huijun Guo, Feng Sang, Hongyu Ma, Hai Peng, Na Zheng, Liran Xu

**Affiliations:** 1 Department of Acquired Immune Deficiency Syndrome Treatment and Research Center, the First Affiliated Hospital, Henan University of Chinese Medicine, Zhengzhou, 450000 China; 2 Key Laboratory of Viral Diseases Prevention and Treatment of Traditional Chinese Medicine of Henan Province, Zhengzhou, 450000 China; 3 Gansu Institute for Drug Control, Yinan Road No. 7, Lanzhou, 730070 China; 4 Thermo Fisher Scientific, Building 6, No. 27, Xin Jinqiao Rd., Pudong, Shanghai 201206 China; 5 Institute for Systems Biology, Jianghan University, Wuhan, Hubei 430056 China

## Abstract

Traditional Chinese medicine (TCM) has been practiced for thousands of years, although concerns about the efficacy, legality, and safety of TCM continue to be raised. Chromatographic studies have detected the presence of heavy metals and plant toxins within some TCM preparations. However, chromatography is not able to identify all of the compounds of TCM, particularly those items that are not clearly labeled on the packaging. The present study aimed to establish a supplemental method that better assesses the ingredient components of TCM preparations.We established an effective approach to screen the biological and toxical composition of TCM based on high-throughput sequencing (HTS), as well as fast detection and validation of the toxical species by real-time PCR, based on ITS2 DNA barcoding. Ruyi jinhuang powder (RHP), a classical herbal prescription containing the toxical herb Arisaematis rhizoma, was chosen to test the method. This method could determine whether the Arisaematis Rhizoma had been replaced by Pinellia pedatisecta in the RHP. The results were validated by real-time PCR. 90% compositions of RHP were identified by ITS2 DNA barcoding, suggesting that more DNA barcoding markers are needed for TCM identification. The strategy of high-throughput sequencing has the potential for comprehensive ingredient profiling for TCM preparations. Real-time PCR provides a expeditious metehod for monitoring the safety and legality of TCM preparations.

## Introduction

Traditional Chinese medicine (TCM) is an important tool for syndrome differentiation, treatment and the clinical application of modern Chinese Medicine. In recent years, safety incidents among traditional Chinese medicine treatments have attracted global attention. The market of Chinese medicinal materials has been found to contain many counterfeits and substitutes, which can affect the healthy development of traditional Chinese medical industry, harm the safety and effectiveness of TCM, and have an impact on the international reputation of TCM. Cases of aristolochic acid nephropathy have led to an international challenge to Chinese herbal medicine and Chinese medicine. European and America media called “have referred to the kidney disease of Chinese herbal medicine” in reference to a ristolochic acid nephropathy. Because of a manufacturing error, one of the herbs in prepared pills (*Stephania tetrandra*) was inadvertently replaced by *Aristolochia fangchi*, which is nephrotoxic and carcinogenic^1^.

The inclusion of counterfeit products and substitutes in Chinese medicinal materials is common. A pharmaceutical market survey found that 123 kinds of popular Chinese medicinal materials had different levels of adulterated products or substitutes^2^. In 2016, Han, J. *et al*. indicated that the market of Chinese medicinal materials contained about 4.2 percent of mixed counterfeit products^3,4^. As of June 2017, searching for the keywords “Chinese medicine pseudoproduct” on the CNKI website (http://www.cnki.net) identified more than 1000 studies on Chinese medicine pseudoproducts. The existence of adulterants and substitutes in the Chinese herbal medicine market is bound to be reflected in the prescribed ingredients of Chinese medicine.

The identification of Chinese traditional patent medcine is more difficult than that of Chinese herbal medicine. Ancient TCM identification technology is limited to the use of the eyes, ears, nose, tongue and other sensory organs to identify the appearance, character and smell of traditional Chinese medicine^5^. Although the identification techniques of TCM were developed significantly already, the 1995 edition of Chinese Pharmacopoeia added the high performance liquid chromatography and gas chromatography to the techniques for identification^6^. No new appraisal methods for identification of TCM were added until the 2015 Chinese Pharmacopoeia^7^. The identification methods of TCM are to be accompanied by the developments of modern science and technology. The traditional identification methods of traditional Chinese medicine are only applicable to the identification of several herbs in the prescription, where the scope of application is limited and the appraisal results depend on the accumulation of professional knowledge and experience, and the professional’s ability to distinguish between species^8^.

Included in the British and Chinese pharmacopoeias^9^, DNA barcoding technology has become the international standard for TCM identification. DNA barcoding technology can perform high-throughput identification of multiple species in mixed samples, and has been used successfully in the study of species composition of animal and plant communities in mixed samples^10,11^. In the present study, we attempted to use high-throughput sequencing (HTS) with DNA barcording to determine the biological composition of a traditional herbal preparation, with the specific goal of detecting the mixed components of traditional patent medicines during industrial preparation. Real time PCR was used to rapidly detect and verify counterfeit and mixed products if they were detected in a different lot of the preparations through HTS. A proper biomarker is important for the quality assessment of TCM preparations via HTS and real-time PCR. The ribosomal internal transcribed spacer 2 (ITS2) has been used as a standard molecular marker to identify medicinal plants for its high inter-specific and intra-specific divergence power^12^. Additionally, the 5.8 S and 28 S rRNA regions, which are located at the two ends of the ITS2 sequence, are conserved enough for primer design^13^.

Ruyi jinhuang powder (RHP) was first mentioned in the Ming dynasty, in Chen Shi-gong’s “*Orthodox of Surgery*”, for surgical common use^14,15^. According to the 2015 edition of the Chinese Pharmacopoeia^7^, Ruyi jinhuang powder is prescribed to detoxify, reduce swelling and relieve pain, use in the hot poison stasis and ulcerative or psoriatic skin. Ruyi jinhuang powder consists of 10 kinds of herbs, including Trichosanthes_kirilowii, Curcuma longa, Glycyrrhiza uralensis Fisch, Angelica dahurica, Phellodendron chinense, Atractylodes lancea, Rheum tanguticum, Citrus reticulate and Arisaematis Rhizoma. Arisaematis Rhizoma is one of the 28 species of toxic Chinese medicinal materials under governmental management. Counterfeit and mixed uses of Arisaematis Rhizoma bring uncertainty to the safe use of Ruyi jinhuang powder. However, in the 2015 edition of the Chinese Pharmacopoeia, no indication of the detection method for the Arisaematis Rhizoma in the Ruyi jinhuang powder is specified^7^. In this study, we chose Ruyi jinhuang powder as the research subject in order to determine the feasibility of using HTS and real-time PCR with DNA barcoding techniques for the identification of prescription ingredients and adulterated products, specifically for the identification of Arisaematis Rhizoma and toxic Chinese medicinal materials.

Prior to testing commercial TCM Ruyi jinhuang powder, we formulated a standard pipeline of quality control with a preparation of known ingredients to verify the reliability and accuracy of the detection method. We randomly selected five medicinal herb sources, covering the root, rhizome, cortex and semen which covered the most common types of compound materials for TCM. The five raw medicinal materials, Panax ginseng (radix and rhizome), Glycyrrhiza uralensis (radix and rhizome), Achyranthes bidentate (radix), Coix lacryma-jobi (semen) and Phellodendron chinense (cortex) included in the composition of the reference sample (REF) were purchased from a local drug store. The purpose of the REF pipeline was not only to identify the components of RHP, but also to establish a detection method for TCM QC. We chose RHP for testing because RHP preparations were composed of 10 types of herbs, and the identification of individual components is difficult. The identification of RHP can reflect the potential of the TCM QC method established by REF. To ensure the accuracy of the reference sample, all materials were identified via DNA barcoding and sanger sequencing, and morphological identification was performed according to the Chinese Pharmacopeia^7^. The sequencing data was added to our reference database for biological ingredient analysis of REF preparations.

## Results

### Establishment of a quality control method for traditional Chinese medicines via high-throughput sequencing

In our study, one reference sample (REF) and one commercial sample of Ruyi jinhuang powder (RHP) were used as the study materials, and the data were acquired through DNA extraction, PCR amplification, library construction and high-throughput sequencing.

**Figure 1:**
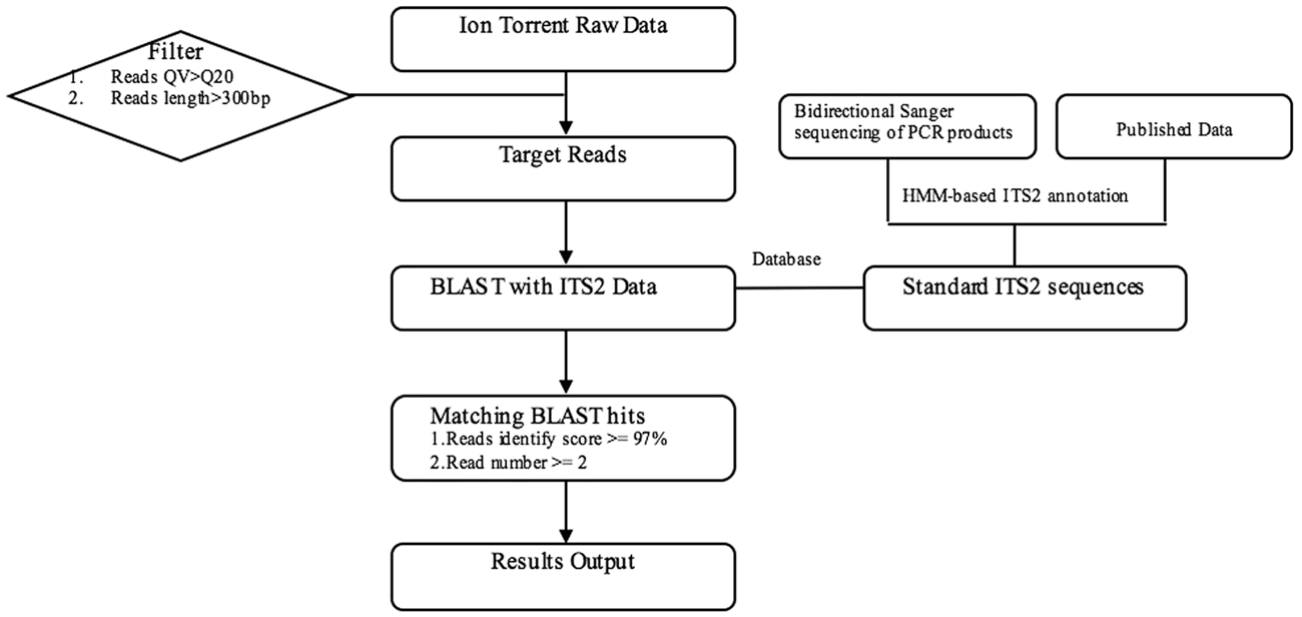
The workflow for the processing and analysis of high-throughput sequencing reads.

A total of 2,029,125 reads, including 603,8215,16 bases, was obtained based on an Ion S5 sequencing platform using the ITS2 biomarker of the reference sample (REF). After filtering adaptors and low-quality sequences (QV < Q20), 1,724,756 (85%) high-quality (HQ) bases with lengths ranging from 50 bp to 500 bp were achieved with a mode read length of 488 bp. After the data quality control procedure (Fig. 1), the Ion S5 sequencer generated 310k trimmed and filtered reads for REF. The filtered reads were then BLASTed with the ITS2 database, which contains no 5.8 s or 28 s rRNA reads. Based on the ITS2 sequences, five prescribed herbal materials were detected in the reference sample, confirming the feasibility of the method for biological ingredient analysis of traditional Chinese medicines. All reads were deposited in the National Center for Biotechnology Information (NCBI) and can be accessed in the Short Read Archive (SRA) under the accession number SRP130186. Sequencing information for each species is summarized in Table 1.

**Table 1:** REF species identification.

Species	Latin name	**C**ounts
Ginseng Radix et Rhizoma	*Panax ginseng* C. A. Me	79943
Achyranthis bidentatae Radix	*Achyranthes bidentata* B1.	157189
Glycyrrhiz Radix et Rhizoma	*Glycyrrhiza uralensis Fisch*.	72834
Coicis Semen	*Coix lacryma-jobi L*. *var*. *ma-yuen* (*Roman*.) *Stapf*	419
Phellodendron chinense Cotex	*Phellodendron chinense* Schneid	20

### Biological ingredient analysis of commercial sample

An in-depth analysis of the species identified in the RHP commercial sample was performed using the same method as for the REF sample. A total of 443,902 reads were obtained based on the ITS2 biomarker of RHP. The mode of the read length was 488 bp. Based on the ITS2 sequences analyzed, eight prescribed raw materials were detected in the commercial sample, whereas Arisaematis Rhizoma and Magnolia officinalis were not found. However, the adulterant species Pinellia pedatisecta and Angelica sinensis were detected (Table 2).

**Table 2:** RHP species identification.

Species	Latin name	Counts
Trichosanthes Radix	*Trichosanthes kirilowii* Maxim	4316
Curcumae longae Rhizoma	*Curcuma Longa* L	764
Glycyrrhiz Radix et Rhizoma	*Glycyrrhiza uralensis* Fisch.	23607
Angelicae dahuricae Radix	*Angelica dahurica* (Fisch ex Hoffm.) Benth et Hook.f.	419
Phellodendron chinense Cotex	*Phellodendron chinense* Schneid	565
Atractylodis Rhizoma	*Atractylodes lancea* (Thunb.) DC	93
Angelicae sinensis Radix	*Angelica sinensis* (*Oliv*.) *Diels*	26
Pedate Pinellia Rhizome	*Pinellia pedatisecta* Schott	4
Rhei Radix et Rhizoma	*Rheum tanguticum* Maxim ex Balf.	3
Citri exocarpium Rubrum	*Citrus reticulata* Blanco	2

### Adulterant cofirmation

In the 2015 edition of the Chinese Pharmacopoeia, there were 17 kinds of TCM compounds containing Arisaematis Rhizoma and processed Arisaematis Rhizoma, but only 3 kinds of TCM were given the microscopic identification methods of processed Arisaematis Rhizoma and no identification methods for Arisaematis Rhizoma were described. In order to verify if Arisaematis Rhizoma was replaced by Pinellia pedatisecta, this study utilized real-time PCR to validate the high homology herbal materials of the commercial Chinese traditional patent medcine. This is the first report to use this method for TCM adulterant component validation.

**Figure 2:**
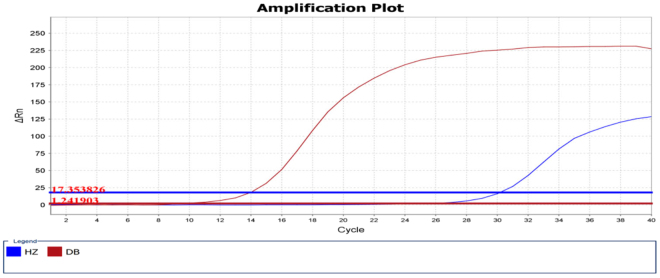
Real-time PCR amplification plots of Arisaematis Rhizoma and Pinellia pedatisecta with specific PCR primers. Blue color of amplification plot is PCR from the amplification of Pinellia pedatisecta with HZ primer and the red amplification plot represents the Arisaematis Rhizoma amplification with DB primer.

**Figure 3:**
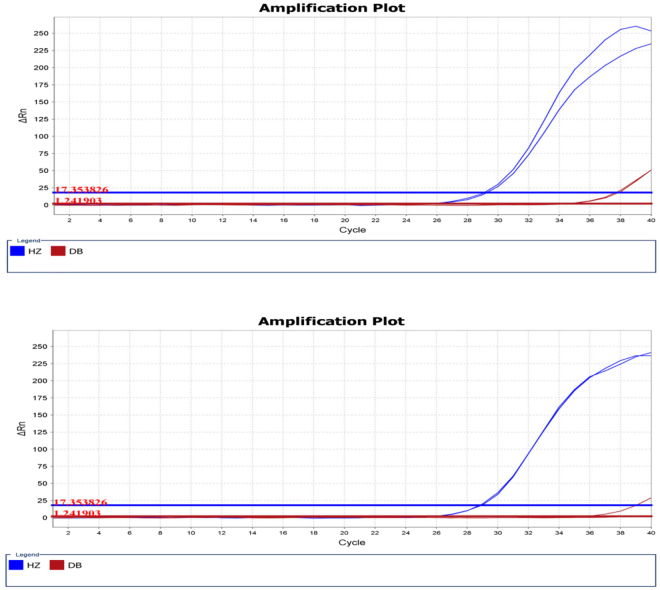
Real-time PCR amplification plots of Arisaematis Rhizoma and Pinellia pedatisecta in Ruyi jinhuang Powder samples from different lots. Blue amplification plot represents PCR from the amplification of Pinellia pedatisecta with HZ primer and the red amplification plot represents the Arisaematis Rhizoma amplification with DB primer.

**Figure 4:**
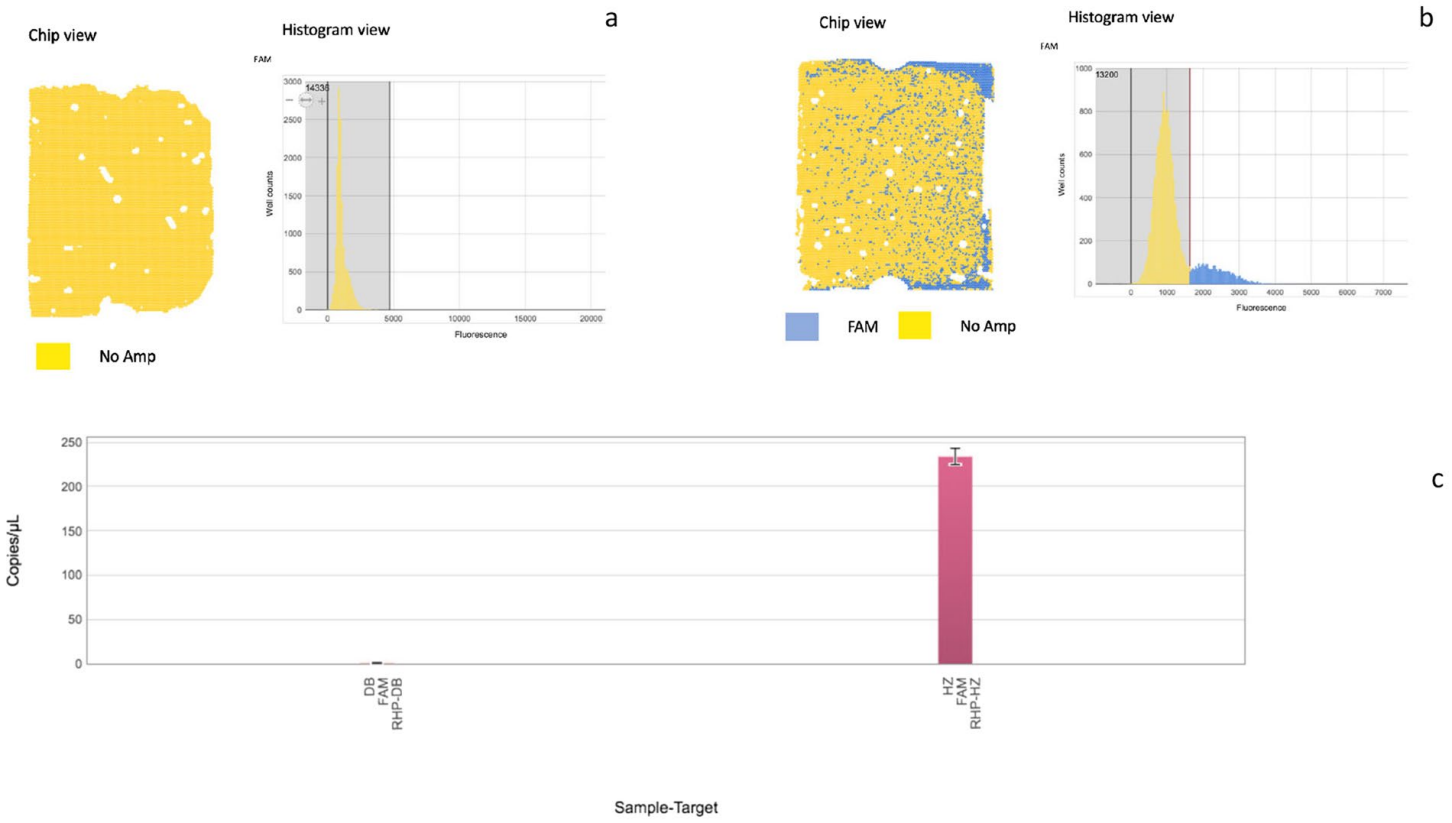
Digital PCR amplification data of quality as displayed in QuantStudio 3D Analysis Suite Software. (**a**) Amplification plot represents the Arisaematis Rhizoma amplification with DB primer in Ruyi jinhuang Powder. (**b**) Amplification results of Pinellia pedatisecta with HZ primer in Ruyi jinhuang Powder. (**c**) Quantification Results of copies of Arisaematis Rhizoma and Pinellia pedatisecta in Ruyi jinhuang Powder. The chip view depicts calls by color, which show an ideal random distribution of the amplified and non-amplified wells (SYBR Green I is read in the FAM channel). The histogram view shows two populations: the larger yellow population corresponds to the non-amplified wells with lower fluorescence, and the smaller blue population corresponds to the amplified wells with significantly higher fluorescence. Good discrimination between non-amplified and amplified populations is observed.

Arisaematis Rhizoma and Pinellia pedatisecta belong to the same genus, and the ITS2 sequence has high homology. We verified the specific real-time PCR primer, and the results showed that the primers can distinguish precisely between Arisaematis Rhizoma and Pinellia pedatisecta (Fig. 2). The primers were used to verifiy the commercial RHP sample. There was no amplification signal of Arisaematis Rhizoma (Ct > 32) in RHP, but amplification curves of Pinellia pedatisecta were present (Ct < 32), which was consistent with the results of high-throughput sequencing. Because high-throughput sequencing is a method better suited for qualitative scanning of components in TCM, we did not use HTS to test specific components of the preparations. Rather, real-time PCR, which is cheaper, faster and more specific, was applied in this role. Another two batches of the commercial Ruyi jinhuang powder (RHP02, Lot No. 16101004; RHP03, Lot No.: 16101006) from the same manufacturer were purchased from the Tong Rentang drug store. These specimens were sampled and tested, using the same method (real-time PCR) applied to the commercial RHP sample. The results for the species in the RHP02 and RHP03 were consistent with the results of RHP (Fig. 3). We also used digital PCR the most sensitivity platform to verify the results, which were in accordance with real-time PCR and HTS (Fig. 4). Real-time PCR primer pairs for each sample is summarized in Table 3.

**Table 3:** Real-time PCR primer pairs for each sample.

No.	Species	Forward Primer (5′ to 3′)	Reverse Primer (5′ to 3′)
**1**	Arisaematis Rhizoma	TGGCCCACCATGCACGT	CGATGAGCATTGTCCACCACT
**2**	Pinellia pedatisecta	TGGCCCACCGTGCACTC	ACGATGAGCGTCCTCCACC

The sequencing results showed that *Magnolia officinalis Rehd*. *et Wils* was not found in the commercial RHP sample. This may be because psbA-trnH is a batter marker for *Magnolia officinalis Rehd*. *et Wils* identification than ITS2. The ITS2 locus is a favorable universal barcode and has stong prospects in authenticating species used across a wide set of plant taxa. Identification efficiency for ITS2 was 92.7% and 99.8% at species and genus level, respectively^12^. The psbA-trnH spacer may be useful as a complementary barcode^12,16,17^.

From the high-throughput sequencing data of RHP, Angelica sinensis, which has high homology with Angelica dahurica, was detected with few reads. The identified score of Angelica sinensis was 100%, suggesting that Angelica sinensis was not a false positive. The methods of Angelica sinensis and Angelica dahurica identification were described in the 2015 edition of the Chinese Pharmacopoeia, and we, therefore, did not develop new method for validation. The purpose of real-time PCR is not to replace the method of identification of the Pharmacopoeia, but as a supplemental method of identification. In the present study, we focused on the detection and verification of toxic medicinal materials, so we verified only the authenticity of Arisaematis Rhizoma.

## Discussion

High-throughput sequencing(HTS) strategy was established in this study to evaluate the quality of traditional patent medicines. HTS appears to be an effective method for monitoring the biological composition of traditional patent medicines. Especially it is an effective approach to screen toxical composition of TCM. Real-time PCR was included to validate the accuracy of identification. This study demonstrates the application of high-throughput sequencing combined with real-time PCR to detect the biological ingredients in herbal preparations. In our study, all of the five prescribed herbal materials in the REF reference samples were detected using ITS2 sequences (Table 1). Based on the ITS2 sequences, 90% prescribed species were detected in commercial sample RHP01 (Table 2). And the method demonstrated that the Arisaematis Rhizoma had been replaced by Pinellia pedatisecta in the RHP. These results were validated by real-time PCR. Fast detection assay of Arisaematis Rhizoma and Pinellia pedatisecta were developed to make up the blank territory of no identification method of Arisaematis Rhizoma of TCM, as given in the 2015 edition of the Chinese Pharmacopoeia. The present study demonstrated the application of sequencing as a high-throughput alternative for the analysis of multiple targets in herbal preparations. Combined with real-time PCR, the high-throughput sequencing is able to detect the biological and toxical ingredients in herbal preparations, and therefore, it can be used for the quality control of traditional patent medicines, particularly considering that the real-time PCR shows strong potential for rapid detection of toxic species in TCM.

Arisaematis Rhizoma, is described as the tuberous rhizome of only three plant species, Arisaema amurense, A. erubescens and Arisaema heterophyllum, in the national pharmacopoeia of China. However, Pinellia pedatisecta are commonly distributed as Arisaematis Rhizoma in medicinal markets because of the similarity of the dried tubers of these plants to those of Arisaematis Rhizoma, as well as their short growing period and high production yield compared to Arisaematis Rhizoma^18^. But Pinellia pedatisecta was not included in the 2015 edition of the Chinese pharmacopoeia, both Pinellia pedatisecta and Arisaematis Rhizoma are toxic Chinese medicinal materials, among which the toxic side effect of the Pinellia pedatisecta is more obvious and the excitant is stronger than the Arisaematis Rhizoma. Counterfeit and mixed uses of Arisaematis Rhizoma not only affects the curative effect of the drug, but also affects the safety of TCM. The 2015 edition of the Chinese Pharmacopoeia did not provide identification method for the Arisaematis Rhizoma of TCM. The real-time PCR used for the validation of Arisaematis Rhizoma and Pinellia pedatiseet make up the blank area.

**Figure 5:**
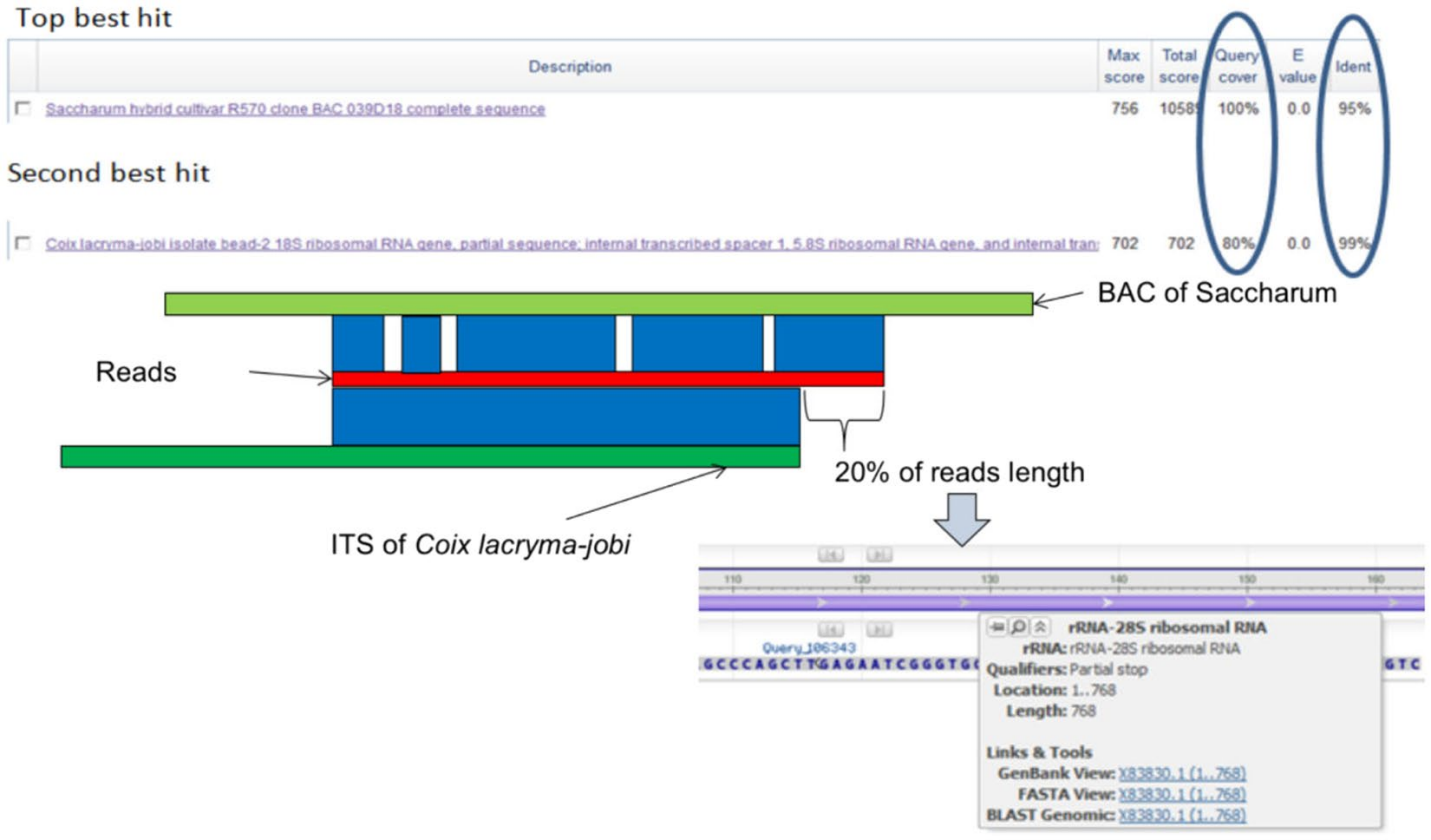
The Annotation of rRNA interfence for the false positive results.

Among molecular sequences, the internal transcribed spacer 2 (ITS2) of the nuclear ribosomal DNA cistron is one of the most frequently used markers for phylogenetics, diagnostics and DNA barcoding^19–22^. At the beginning of our data analysis, we did not acquire strongly convincing analytical results. The Ion Torrent sequencing data directly BLASTed the data with Nr databse, resulting in dozens of false positive results. In order to resolved the false positive interference, we referred to some TCM barcoding classical papers to optimize our data analysis pipeline^10,23,24^. We increased the filter criteria in the analysis pipeline, adding a read length cut-off of 300 bp (reads shorter than 300 bp were filtered). The average length of ITS2 is 160–320 bp^12^. However, ITS2 has a primer length of about 80 bp so we were able to get as much of the full length of ITS2 as possible if the read length was longer than 300 bp. Meanwhile, we increased the quality criteria, selecting reads quality value greater than Q20, and used BLAST to obtain the output of reads that had 2 or more identified species. The identified score was 97%, which was derived from the clustering criteria in the OTU metagenomics clustering analysis^25^. Based on the combination of the ITS2 sequences, five prescribed species were detected in REF, but we obtained a false positive identification for the species Saccharum. More than 500 reads were compared to Saccharum. We performed high-throughput sequencing based on the ITS2 amplicons for Panax ginseng, Glycyrrhiza uralensis, Achyranthes bidentat, Phellodendron chinense and Coix lacryma-jobi in order to determine from which species of Saccharum the results came. The sequencing data were processed and analyzed according to the previous analysis pipeline. Finally, we found that 94% of the reads of Coix lacryma-jobi were identified as Saccharum. We randomly picked some of the reads to BLAST against the NCBI Nr database. The best match was Saccharum, with a query cover score of 100%, and identified score of 95%. The next best hit was Coix lacryma-jobi, with a query cover score of 80% and identified score of 99%. We BLASTed the 20% of the reads that did not match Coix lacryma-jobi and obtained rRNA-28s as the result (Fig. 5). This result was important, because it suggested that the Saccharum false-positives appeared mostly due to 28 s rRNA interference. Therefore, we optimized the data analysis process and replaced the alignment database with HMM-based annotation^13^. In order to resolved the false positive interference, we built an accurate and clean ITS2 database. The ITS2 Database presents an exhaustive dataset of internal transcribed spacer 2 sequences from published work^12,26,27^. Following an annotation by profile Hidden Markov Models (HMMs), all of the clean ITS2 sequences without 5.8 s and 28 s rRNA were inserted in an ITS2 database. Hundreds of accurate and clear ITS2 sequences referred to the book of *Standard DNA barcodes of Chinese materia medica in Chinese pharmacopoeia* added to our database as reference reads. Without the interference of 28 s rRNA, 533 of the false-positive reads were aligned to Coix lacryma-jobi. The 5 species in the reference sample were identified with 100% certainty. The ITS2 database without 5.8 s or 28 s rRNA sequences was the key of the analysis, because 5.8 s and 28 s rRNA are highly conserved and false-positive results can be readily introduced into the analysis. In general, several dozen species were generated in REF before the analysis filter was applied, and false positive results were excluded. Standard ITS2 reference reads excluding 5.8 s and 28 s rRNA false positive interference were excluded. High quality reads (AQv > 20), long read sequences (reads shorter than 300 bp were filtered) and high identify score (identify score > 97%) were also used to filter out false positive interference caused by low quality reads. HTS is very sensitive, and avoiding contamination of the samples is of primary import. In general, the DNA extraction, library building and PCR amplification must follow the guidelines of “Laboratory management methods of clinical gene amplification test in medical institutions”. Strict experimental zoning is an important measure to eliminate contamination. In particular, DNA extraction and ITS2 amplification required particular attention to sterile technique. Good wet lab produces high quality data.

The most frequently used methods for chemical constituent analysis of TCMs are various chromatographic and spectroscopic methods^28,29^. However, these targeted approaches only measure the chemicals of interest, and are not capable of assessing contaminant ingredients. Contaminant species refer to ingredients not listed on the package, which are usually considered useless for efficacy, reduce the efficacy or even cause side effects. In the 2015 edition of the Chinese Pharmacopoeia, there is no proscribed method of appraisal for Arisaematis Rhizoma in Ruyi jinhuang powder^7^. In this study, we were not only able to determine that there was no Arisaematis Rhizoma present in the RHP sample, but also that Pinellia pedatisecta had been substituted. The development of high-throughput sequencing technologies is key to realizing identification of ingredients Chinese medicinal compounds. Currently, the most wildly used high-throughput sequencing platforms are the Ion Torrent and Illumina Hiseq series platforms. The Illumina HiSeq. 2000 features greater throughput, but the short read lengths remain a major shortcoming. Moreover, a complete run requires 3–10 days. Compared with the Illumina sequencers, the Ion Torrent has several advantages. First, sample preparation is rapid, requiring only 4 h instead of days. In addition, a sequencing reaction on the Ion S5 sequencer requires only 120 min. The average length of a single read is 600 bp, which is longer than that of the Illumina platform, and the long reads have high consensus accuracy, which is not significantly different with Illumina generated data^30^. Third, the cost of the amplicon sequencing on Ion Torrent platform is cheaper than on the Illumina platform because no lasers, optics, camera or enzyme cascade is necessary in the sequencing processing. Relative to the costs associated with the Illumina sequencing platforms, the Ion Torrent sequencing instruments are cheaper. Moreover, using Ion Torrent sequencers can greatly shorten the time required for detecting and analyzing samples. Based on the results of this appraisal, we developed an automated analysis plugin-TCM v1.0, paired with the Ion Torrent platform, which can analyze the high-throughput data automatically. From the sample preparation to the identification results, the entire workflow needs no more than 24 h. The application of the Ion Torrent sequencing platform to biological component testing of TCM will improve the sensitivity of this approach.

High-throughput sequencing has several potential limitations. First, high-throughput sequencing cannot be performed effectively in the presence of DNA degradation that arises from the processing of traditional patent medicines, because DNA degradation affects PCR amplification of DNA marker^31^. DNA barcoding cannot quantitatively measure the elements in traditional Chinese medicine preparations, because the efficiency of DNA extraction of each medicinal component and the amplification of DNA markers directly affects the final read output and there is no correlation between a compound and the number of reads. But with the development of technology, this limitation may be solved. The third generation of PCR technology-digital PCR, which can reach the detection sensitivity of 0.01%, is based on a new algorithm^32^. With digital PCR, there is no need to consider the amplification efficiency of PCR, which provides the possibility for the quantitative provision of compound components.

In the present study, we pioneered the use of real-time PCR to verify our results, which were consistent with the sequencing results. This shows that real-time PCR is a good complement to high-throughput sequencing. In light of the two potential shortcomings of high- throughput sequencing method for the identification of TCM compounds, namely DNA degradation resulting in false negatives and the inability of HT sequencing to quantify component composition, real-time PCR is a suitable supplemental method. The quality requirement of DNA for real-time PCR amplification is not high, so long as the amplicon length is shorter than the length of the DNA. Many preparations of TCM compounds are processed, which can degrade the DNA and result in failed identification by sanger and high-throughput sequencing methods^31^. However, real-time PCR can be employed to solve the problem of DNA degradation.

There are several papers published in recent years, which have discussed the quality control of traditional patent medicine using SMRT Sequencing and DNA Barcoding. They were cited by our study, but in the current paper, we established a holistic solution for TCM QC. There are no similar articles in the TCM QC articles published thus far. NGS has the ability to discover unknown additions or substitutes in compounds. However, NGS is expensive, has a high technical requirement and is not fast enough for convenient use. Real-time PCR is cheaper, faster and specific, and is well suited for validation and fast detection of unknown additions or substitutions in the compounds found by NGS. NGS combined with real-time PCR is the innovation of this study. In our study, the specific PCR method (SPM) confirmed that one of the herbs in the commercial pills (Arisaematis Rhizoma) was replaced by Pinellia pedatisecta. This method can be used to identify different species, and is especially useful detecting toxic and valuable medicinal materials in the compound. Because the real-time PCR method is inexpensive and rapid, it presents an efficient method for the rapid detection of toxic materials in preparations. In future studies, we will aim to increase the real-time PCR data of different species to evaluate the sensitivity and specificity of this method.

In conclusion, we established an effective approach for monitoring the biological composition of traditional Chinese medicines based on high-throughput sequencing and DNA barcoding. Real-time PCR was included to validate the accuracy of identification. This study demonstrates the application of high-throughput sequencing combined with real-time PCR to detect the biological and toxical ingredients in herbal preparations. In the near future, this method may become a powerful tool for the quality control of traditional patent medicines, particularly considering that real-time PCR shows strong potential for applications of rapid detection of toxic species in TCM.

## Materials and Methods

### Sample collection

Five raw materials composing the reference sample were purchased from Tong Rentang drug store, including Panax ginseng (radix and rhizome), Glycyrrhiza uralensis (radix and rhizome), Achyranthes bidentate (radix), Phellodendron chinense (cortex) and Coix lacryma-jobi (semen). To ensure the accuracy of the reference samples, all materials were identified via the DNA barcoding and Sanger sequencing, and morphological identification was performed according to the Chinese Pharmacopeia^7^. The materials were crushed into powder and marked as REF. Commercial Ruyi jinhuang powder specimens were randomly purchased from Tong Rentang drugstore (Lot No.: 3101027). The commercial Ruyi jinhuang powder was marked as RHP.

### DNA extraction and quantification

Total genomic DNA was isolated using the PureLink™ Genomic Plant DNA Purification Kit (part #, K183001, Thermo Fisher Scientific Inc., Waltham, MA, USA), according to the manufacturer’s instructions. Briefly, 30 mg of the sample was weighed and transferred into a 1.5 ml centrifuge tube. Lysis buffer and Proteinase K from the kit were added, followed by vortexing for 1 min and pulse spinning. The tube was incubated at 56 °C for 1 h with occasional swirling. The other steps were the same as outlined in the previously published protocol. The obtained DNA concentration was quantified on a NanoDrop 2000 spectrophotometer and Qubit 3.0 (Thermo Fisher Scientific).

### PCR Amplification and Purification

PCR amplification was performed according to the DNA barcoding protocol in the Chinese Pharmacopoeia on an Applied Biosystems Veriti^TM^ Thermal Cycler (Thermo Fisher Scientific). Individual amplifications of ITS2 were carried out in 25 uL total volumes, including 10 ng template DNA and 2× Master Mix (AmpliTaq gold fast PCR MM, Thermo Fisher Scientific). The PCR annealing temperature was increased to 58 °C, and 25 cycles were run.

The PCR products were purified with Agencount Ampure XP beads (Beckman) and the PCR size and concentrations were measured on an Agilent 2100 Bioanalyzer (Agilent Technologies, Inc., Folsom, CA, USA) with the Qubit platform.

### Library construction and Sequencing

ITS2 amplicon sequencing was performed using the Ion Torrent platform (Thermo Fisher Scientific). The purified PCR amplicons were used to construct an amplicon library using the Ion Plus Fragment library kit (part # 4471252, Thermo Fisher Scientific). After library construction, quality control was carried out on Agilent 2100 Bioanalyzer and quantified with Ion Library quantification kit (part # 4468802, Thermo Fisher Scientific). Template preparation was performed using the Ion OneTouch System (Thermo Fisher Scientific) with the Ion 520 kit-OT2 (part # A27751, Thermo Fisher Scientific). Templates were sequenced on an Ion S5 sequencer. The overall quality of each run was evaluated based on the report generated by the Torrent Server. Quality filters for downstream bioinformatics analysis included: (1) At least 10,000 reads per sample and (2) 99% of targeted positions covered at 1, 97% at 20, and 95% at 100 (data generated by Torrent Suite). The median read length was 500 bp.

### Sequencing Data analysis

Using the Ion S5 sequencing platform, high-quality sequences (>99% accuracy on single base reads) were selected for further processing and BLAST analysis. A subsequent filtering step included the masking of ITS2 PCR primer sequences and sequences shorter than 300 bp were removed before BLAST analysis. The TCMV1.0 plugin (provided with the Ion Torrent sequencer) was used for sequence filtering and BLAST, using the default parameters.

### Real-time PCR analysis

DNA was extracted from the powder of the herbal martials and commercial patent medicines using a PureLink™ Genomic Plant DNA Purification Kit (part #, K183001, Thermo Fisher Scientific). The DNA was diluted 10-fold with deionized water before use as a template for real-time PCR. Each reaction contained 10 ul 2× PowerUp SYBR Green Master Mix (part # A25742, Thermo Fisher Scientific). 1 uM each of the forward and reverse primers, and 1 ul of template DNA. The total reaction volume was 20 ul. PCR amplification was performed under the following conditions: 95 °C for 30 s, followed by 40 cycles at 95 °C for 5 s and 60 °C for 30 s using a ViiA7 Real-Time system (Thermo Fisher Scientific). All the qRT-PCR analyses were performed three times with independent DNA samples.

### Digital PCR analysis

Sample preparation, chip loading, and thermal cycling were performed using the standard conditions recommended in the user’s manual. To prepare the digital PCR master mix, SYBR Green I dye (part #, S7567, Thermo Fisher Scientific) was diluted to 20× in TE buffer at pH 8.0. The 20× stock in TE was discarded after use. The reaction mix was prepared by adding: 7.25 μL QuantStudio 3D Digital PCR Master Mix (part #, 4482710, Thermo Fisher Scientific), 1.45 μL 20× SYBR Green I dye in TE buffer (pH 8), 200 nM each of forward and reverse primers, 10–50 ng total DNA sample, and enough water to bring the volume to 14.5 μL. 14.5 μL of reaction mix was loaded onto the QuantStudio 3D Digital PCR Chip (part #, A26316, Thermo Fisher Scientific) using the chip loader, and run using the standard thermal cycling conditions recommended in the user’s manual. The prepared chips were analyzed using the QuantStudio 3D Digital PCR Instrument (part #, 4489084, Thermo Fisher Scientific). SYBR Green I dye was read in the FAM dye channel due to its similar spectral properties.
